# Editorial: Natural product treatment of gastrointestinal diseases

**DOI:** 10.3389/fphar.2022.1074528

**Published:** 2022-11-09

**Authors:** Mingyu Sun, Hailian Shi, Hemant Goyal

**Affiliations:** ^1^ Institute of Liver Diseases, Shuguang Hospital Affiliated to Shanghai University of Traditional Chinese Medicine, Shanghai, China; ^2^ Shanghai University of Traditional Chinese Medicine, Shanghai, China; ^3^ University of Texas Health Science Center at Houston, Houston, TX, United States

**Keywords:** natural product, intestinal flora, inflammation and immunity, gastrointestinal diseases, herbal formulae

Gastrointestinal (GI) cancers, including esophageal, gastric, and colorectal cancers, are one of the major malignant diseases detrimental to health and account for almost 20% of all cancers worldwide. Besides their high incidence, GI cancers are related to high mortality rates, placing these malignancies among the most prominent public health issues of our time. Gastrointestinal diseases are prevalent, especially functional gastrointestinal disorders. Natural product has been used for thousands of years in the treatment of gastrointestinal diseases including cancer. To discover and develop the natural product with therapeutic selectivity and without toxicity are very important steps in the treatment of GI diseases. Because of their wide range of pharmacologic activities and low toxicity in animal models, many natural products have been used as alternative treatments for gastrointestinal diseases and cancers.

This Research Topic aimed at widening the knowledges on natural product treatment of Gastrointestinal Diseases. The articles received by the journal were carefully reviewed, thus offering a high-quality Research Topic. We summarize the main findings and perspectives detailed within each of the nine accepted articles.

Colorectal cancer (CRC) is the third most common cancer worldwide. Despite the improved knowledge on CRC heterogeneity and advances in the medical sciences, it is still urgent to cope with the challenges and side effects of common treatments for the disease. Actinobacteria are known to be prolific producers of a wide range of bioactive natural products against CRC. This review by Bahrami et al. is a holistic picture on actinobacter-derived cytotoxic compounds against CRC. This review describes the chemical structure of 232 natural products presenting anti-CRC activity with the being majority of quinones, lactones, alkaloids, peptides, and glycosides. Most of these natural products are derived from marine actinobacteria followed by terrestrial and endophytic actinobacteria, respectively. The high diversity of actinobacterial strains and their natural products derivatives, described here provides a new perspective and direction for the production of new anti-CRC drugs and paves the way to innovation for drugs discovery in the future.

Previous researches proved the inhibitory effect of quercetin on breast cancer, ovarian cancer and gastric cancer, but the inhibitory effect of quercetin on colorectal cancer still needs to be clarified. Chen et al. proved that quercetin inhibited the tumorigenesis of CRC cells through downregulating hsa_circ_0006990. Quercetin induced the differentiation of M2-TAMs into M1-TAMs by inhibiting autophagy and then downregulated hsa_circ_0006990 in CRC cells co-cultured with M2-TAMs, finally inhibiting the proliferation and metastasis of CRC cells.

More and more research evidences have proved that gut microbiota plays a very important role both in the pathological process of gastrointestinal diseases and gastrointestinal cancers. Researchers are increasingly concerned about the role of gut bacteria in natural products to treat gastrointestinal diseases. How to regulate the intestinal flora and the advantages of the action of natural products is a growing topic of interest. Lu et al. review paper summarizes the role of gut microbiota in colorectal tumorigenesis and the mechanism by which natural products reduce tumorigenesis and improve therapeutic response.

However, gut microbiota also affected the serum metabolism of natural products. Cai et al. found that antineoplastic Fufangchangtai (FFCT) showed no significant effect on the tumor volume of colorectal tumor-bearing nude mice, but could increase the levels of CD4^+^ and CD8+T lymphocytes. Furthermore, the tumor induced dysbiosis of gut bacteria could affect the absorption and metabolism of FFCT, evidenced by the decreased levels of Matrine, Isogingerenone B and Armillaripin in tumor-bearing mice after FFCT intervention. And, FFCT could increase abundance of Roseburia, Turicibacter and Flexispira, indicating that FFCT might improve the intestinal microenvironment by modulating gut microbiota in colorectal tumor-bearing mice.

Esophageal squamous cell carcinoma (ESCC) is one of the deadliest digestive system cancers worldwide lacking effective therapeutic strategies. Chen et al. explored that the natural product celastrol coordinatively triggered extrinsic and intrinsic apoptosis pathways to diminish the tumor growth of ESCC *in vivo* and *in vitro*, by synergistically triggering DR5-dependent extrinsic apoptosis and Noxa-dependent intrinsic apoptosis through transactivation of ATF4 in ESCC cells. Furthermore, FoxO3a-Bim pathway was also involved in the intrinsic apoptosis of ESCC cells induced by celastrol. The findings highlighted celastrol as a promising apoptosis-inducing therapeutic strategy for ESCC.

Irritable bowel syndrome (IBS) is characterized by recurrent abdominal pain associated with disordered defecation. Jiang et al. revealed Wenshen-Jianpi prescription can alleviate the visceral hypersensitivity in IBS-D model rats, mainly accompanied by down regulating the expression of TNF-α, p-MEK1/2, p-ERK1 and p-ERK2 in the colon tissue.

Inflammatory bowel diseases (IBDs) are systemic diseases and not only impair the gut functions but also influence extraintestinal organs, such as the liver, kidney, and eyes. Yu et al. contributed their research paper that Cao-Xiang-Wei-Kang capsule alleviates the progression of murine experimental colitis induced by dextran sodium sulfate (DSS) by suppressing inflammation, promoting mucosal healing, and re-establishing a microbiome profile that favors re-epithelization.

Pterostilbene (PTE) is a natural polyphenol compound that has been proven to improve intestinal inflammation, but its laxative effect on slow transit constipation (STC) has never been studied. Yao et al. presented a research paper that PTE might alleviate constipation by inhibiting oxidative stress-induced apoptosis of interstitial Cajal cells through modulating PI3K/AKT/Nrf2 signaling pathway. PTE also attenuated the dysbiosis of gut microbiota in STC mice.

Acute pancreatitis (AP) is one kind of inflammatory diseases of the pancreas with early local symptoms and followed by systemic inflammatory response. Progression of AP into severe acute pancreatitis (SAP) usually leads to a life-threatening condition with multiple organ dysfunction, particularly acute lung injury (ALI), which is the cause of most deaths during the early phase of AP. A study by Wu et al. explored that Emodin has a therapeutic effect on AP-associated lung injury, which is at least partially due to the inhibition of NLRP3/Caspase1/GSDMD-mediated AMs pyroptosis signaling ways.

